# Intergenerational Transmission of Characters Through Genetics, Epigenetics, Microbiota, and Learning in Livestock

**DOI:** 10.3389/fgene.2019.01058

**Published:** 2019-10-31

**Authors:** Ingrid David, Laurianne Canario, Sylvie Combes, Julie Demars

**Affiliations:** GenPhySE, Université de Toulouse, INRA, ENVT, Castanet Tolosan, France

**Keywords:** non-genetic inheritance, genetic, epigenetic, microbiota, culture, behavior, livestock

## Abstract

Evolutionary biologists studying wild species have demonstrated that genetic and non-genetic sources of information are inherited across generations and are therefore responsible for phenotypic resemblance between relatives. Although it has been postulated that non-genetic sources of inheritance are important in natural selection, they are not taken into account for livestock selection that is based on genetic inheritance only. According to the natural selection theory, the contribution of non-genetic inheritance may be significant for the transmission of characters. If this theory is confirmed in livestock, not considering non-genetic means of transmission in selection schemes might prevent achieving maximum progress in the livestock populations being selected. The present discussion paper reviews the different mechanisms of genetic and non-genetic inheritance reported in the literature as occurring in livestock species. Non-genetic sources of inheritance comprise information transmitted *via* physical means, such as epigenetic and microbiota inheritance, and those transmitted *via* learning mechanisms: behavioral, cultural and ecological inheritance. In the first part of this paper we review the evidence that suggests that both genetic and non-genetic information contribute to inheritance in livestock (i.e. transmitted from one generation to the next and causing phenotypic differences between individuals) and discuss how the environment may influence non-genetic inherited factors. Then, in a second step, we consider methods for favoring the transmission of non-genetic inherited factors by estimating and selecting animals on their extended transmissible value and/or introducing favorable non-genetic factors *via* the animals’ environment.

## Introduction

For a long time, the transmission of DNA sequence from one generation to the next was considered as the only lever explaining evolution and natural selection. But more recently, several scientists demonstrated that non-genetic information that can cause phenotypic differences between animals can also be inherited across generations. In response to this finding, evolutionary biologists have developed the concept of inclusive or general heritability that combines all sources of information inherited across generations [for a review of the different genetic and non-genetic sources of inheritance, see Mameli (2004) and Danchin et al. (2011)]. Distinguishing between these different additional inheritances is sometimes difficult (Rossiter, 1996). Nonetheless, they can be classified into two main categories based on the means of transmission. 1) The inherited information is transmitted physically from one generation to the next. This is the case for epigenetic marks (epigenetic inheritance) and other media such as metabolites and symbionts (microbiota inheritance). 2) The information is not transmitted physically as is the case for environmental inheritance that can be further divided into behavioral (Jablonka and Lamb, 2014), cultural (Feldman and Cavalli-Sforza, 1975; Danchin et al., 2011) and ecological inheritance (Odling-Smee et al., 2003). The importance of epigenetics in mammalian and plant characters has been emphasized in numerous studies (reviewed in Charlesworth et al. (2017) and in Quadrana and Colot (2016) for plants) and the vertical transmission of some epigenetic marks has been demonstrated (Heard and Martienssen, 2014; Van Otterdijk and Michels, 2016). The microbiota consists of the symbiotic microbial cells (bacteria, archaea, viruses, and eukaryotic microbes) that reside within and on the body of animals. The vertical transmission of the microbiota has been described in various species (Sonnenburg et al., 2016; Sandoval-Motta et al., 2017), as has its impact on the physiology of the host (Sommer and Bäckhed, 2013; Marchesi et al., 2015). Environmental inheritance is defined as the information that passes from one individual to another *via* learning mechanisms or transmission of environmental conditions. Learning from conspecifics or adaptation to the environment may occur through observation, imitation, teaching or interactions in the form of play behavior, aggressive encounters, cooperation, or competition to access a resource (Nicol, 1995).

These different sources of inheritance were described in evolutionary studies to understand the mechanisms that drive natural selection (Mameli, 2004). Among others, the author tells the story of the lucky butterfly to illustrate the importance of non-genetic inheritance in natural selection: the non-genetic inherited laying preference (new or old plant) in a genetically identical butterfly population resulted in the extinction of the sub-population laying in the old plant. Even if discussed (Goddard and Whitelaw, 2014), non-genetic sources of inheritance are not currently taken into account in livestock selection strategies that are based on genetic inheritance only, the animals to be the parents of the next generation being selected on the basis of their breeding values for the characters of interest (Fisher, 1918). This type of genetic selection has proven to be efficient, with for instance much progress achieved for a number of production characters in different livestock and plant species (reviewed for farm animals in Rauw et al. (1998)). Nonetheless if, as suggested in evolutionary studies, the non-genetic inheritance may be important for the transmission of characters (Mameli, 2004; Heard and Martienssen, 2014), then not considering non-genetic inheritance in selection schemes may be an obstacle to achieving maximum progress in livestock populations (David and Ricard, 2019).

The objective of this discussion paper is to invite readers thinking about additional transmitted effects different from genetics. Our aim is not to explain in detail, as in David and Ricard (2019), how to decipher these different sources of inheritance statistically. The first part of the paper reviews the different mechanisms of inheritance in livestock. The second part makes practical suggestion that may help increase the benefit of selective breeding by accounting for non-genetic inheritance.

## The Different Sources of Heritability in Livestock Species

**Figure 1:**
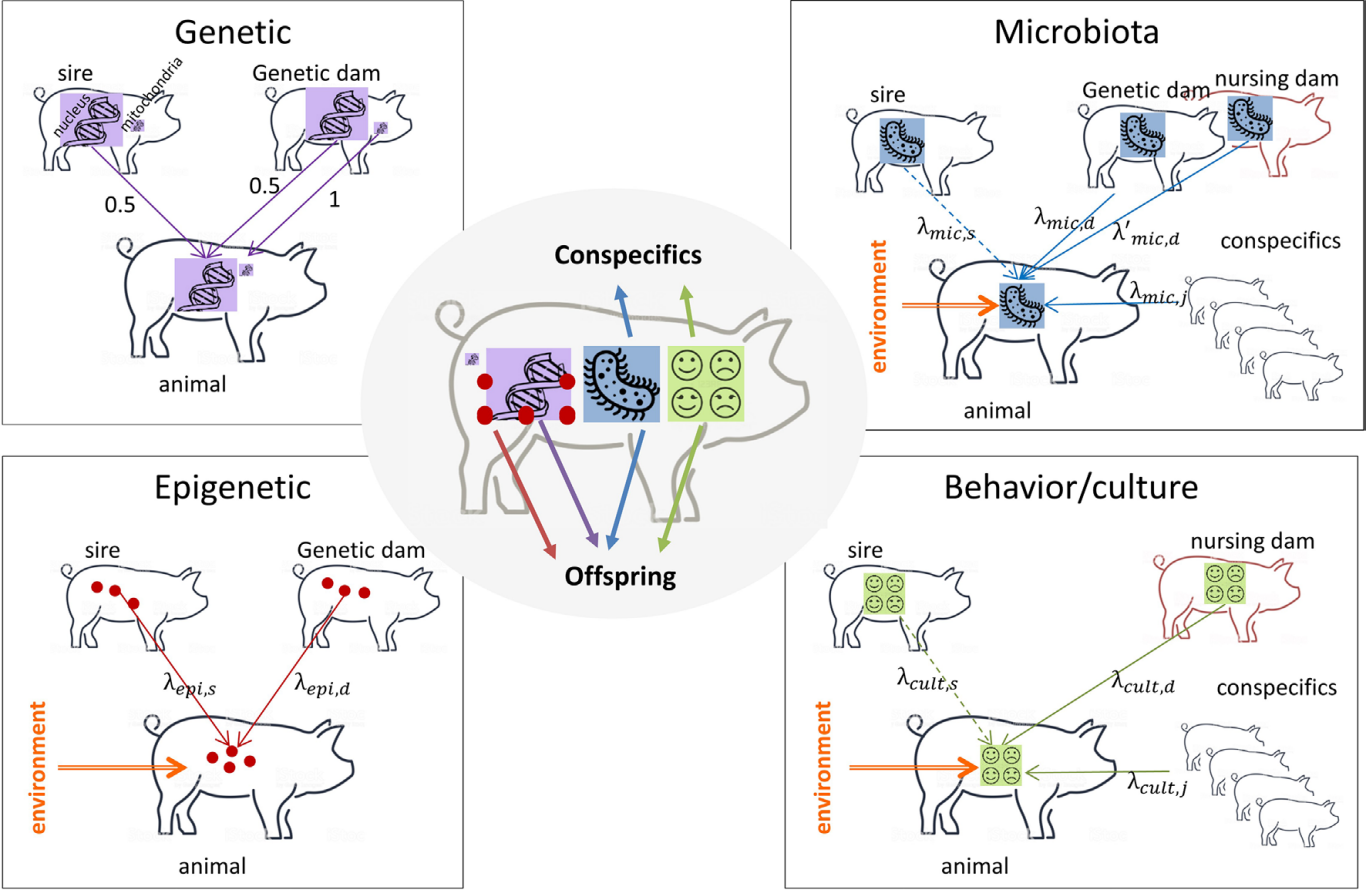
What an animal receives and transmits. Purple arrows: DNA transmission; red arrows: epigenetic mark transmission; blue arrows: microbiota transmission; green arrows: behavior/culture transmission; orange arrows: environment that modifies the transmitted information support. Dotted lines: transmission for specific types of livestock only when the sire is in contact with his offspring (meat sheep, meat cattle). *Genetic*: sire and genetic dam transmit half of their DNA to their offspring (with the particularity of sex chromosomes X and Y that are not identical and mitochondrial DNA that is transmitted by the dam only). *Epigenetic*: sire and genetic dam transmit part of their epigenetic marks to their offspring, epigenetic marks are modified by the environment. *Microbiota*: genetic and nursing dams transmit part of their microbiota to the offspring, conspecifics can share their microbiota with the focal individual. The sire, if present after delivery, can transmit its microbiota to his offspring. Microbiota is modified by the environment. *Behavior/culture*: the nursing dam transmits part of her behavior to the offspring, the focal individual can learn from conspecifics and from its sire if present after delivery. Behavioral traits can be modified by the environment (stressful situations for instance). An animal transmits all factors to its offspring, microbiota and culture to conspecifics.

Selection is efficient if applied on factors that are heritable. A heritable factor is a factor that is stable across generations (i.e. inherited) and that causes phenotypic differences between individuals of a population (Mameli, 2004) leading to similarity between relatives. The transmission of the different inherited factors is described in **Figure 1**. The mean of transmission is different and has nothing to do with the mean of action of a factor that corresponds to its (direct or indirect) influence on the phenotype of an individual (see end of section 2 for an explicit distinction).

### Information Physically Inherited Across Generations

#### Genetic Inheritance

It is well established that DNA is transmitted from one generation to the next through the sexual reproduction process (meiosis). Offspring receive one half of their DNA from each parent apart from mitochondrial DNA that is transmitted by the dam only (Gyllensten et al., 1985). Based on his experiments, Mendel established the laws of inheritance (law of segregation and independent assortment) describing how genes are transmitted from one generation to the next. The DNA is thus inherited.

It is also well known that the DNA is the support of gene expression through the transcription process that transcribes the DNA to produce a molecule of RNA and the translation process during which the mRNA sequence is decoded to specify the amino acid sequence of a polypeptide. Recent advancements in high-throughput technologies have resulted in the measurement of multiple types of high-dimensional omics data (genomics, transcriptomics, and metabolomics) in order to better comprehend the relationship between genotype and phenotype (Cavalli-Sforza and Feldman, 1973; Safo et al., 2018). Numerous studies have described how different alleles lead to different phenotypes and it has long been considered that the maternal and paternal alleles received by the offspring are expressed in a dominance, incomplete-dominance, and co-dominance manner, the phenotype being the result of these expressions. To name just one example, it has been demonstrated in dairy breeds that a mutation (substitution) in the *DGAT1* gene induces a modification of its expression which has a major effect on the milk fat content and other milk characteristics (Grisart et al., 2002; Grisart et al., 2004). Nonetheless, some genes do not follow this rule of co-expression: imprinted genes display mono-allelic expression and this allele-specific regulation is entirely dependent on whether the gene is inherited from the dam or the sire (Reik and Lewis, 2005). In livestock species, the essential role played by imprinted genes has been demonstrated through the investigation of the genetic architecture of performance characters. Two mutations in imprinted genes have been identified and are associated with muscular hypertrophy: a mutation within the paternal *IGF2* gene in pigs (Van Laere et al., 2003) and a mutation within the paternal *DLK1* gene in sheep (Freking et al., 2002). The *IGF2* variant displayed a typical imprinting effect. Both homozygous individuals for the mutation (patIGF2^mut^|matIGF2^mut^) and heterozygous individuals for the mutated allele carried by the paternal chromosome (patIGF2^mut^|matIGF2^wt^) show hypermuscularity (Van Laere et al., 2003). A more complex way of imprinting, called polar overdominance, is illustrated by the *DLK1* mutation in sheep (Cockett et al., 1998). In this example, only individuals that have inherited the mutated allele from their sire (patDLK1^mut^|matDLK1^wt^) express the phenotype of muscular hypertrophy (Freking et al., 2002).

To sum up, genes are inherited by the transmission of genetic material, DNA, and their expression plays an important role in the development of all phenotypes. Genetic inheritance thus contributes to phenotypic resemblance between relatives.

#### Epigenetic Inheritance

The epigenome consists of all the molecular processes that interact with the genome of an organism and contribute to the regulation of gene expression without modifying the DNA sequence. Epigenetic modifications include DNA methylation or hydroxymethylation of CG dinucleotides, chemical modifications of histones, interactions between the DNA and small RNAs, and different states of chromatin condensation (Feil and Fraga, 2012). Epigenetic mechanisms act as possible mediators of the response of an individual to modifications of its environment. Non-genetic transmission across generations could happen at several time scales. Firstly, *in utero* exposure of developing embryos may directly impact phenotypes at birth and later on of individuals of the first generation (F1); it is called intergenerational transmission. Secondly, the prenatal exposure of fetuses may alter their gametes and affect performances of offspring produced in the second generation (F2); it corresponds to a multigenerational transmission. Thirdly, a phenotypic effect persists in individuals of the third generation (F3) while a modification of the environment occurred only on F1 embryos and gametes; this is a true transgenerational inheritance. In addition, if a male or non-pregnant female adult animal is subjected to an environmental exposure, then changes seen in the F2 generation or later are considered transgenerational.

It is now well established that epigenetic information is transmitted from one generation to the next one. Various studies have assessed the effect of environmental factors on the phenotype through epigenetic mechanisms by evaluating how a modification of the environment affects the phenotype. In mice, increased methyl-group donor intake during pregnancy affected the coat color phenotype of offspring (Dolinoy et al., 2006). Hypermethylation of the transposable element in the agouti gene led to its expression silencing and resulted in a brown coat color (Dolinoy et al., 2006). In livestock, many studies performed in cattle, pigs, sheep, and chicken have highlighted direct effects of exposure to environmental toxicants and nutrients (reviewed in Feeney et al., 2014) on the phenotypes. For example, in pigs, the consequences of maternal dietary protein content on the transcriptional regulation of the myostatin (MSTN) gene in the skeletal muscle of the offspring was determined (Liu et al., 2011). The maternal diet affected MSTN expression in exposed offspring *via* epigenetic mechanisms such as histone modifications and microRNA expression (Liu et al., 2011). Maternal behavior modulating the developmental environment of the offspring is also responsible for inducing epigenetic marks in the offspring, thus contributing to the epigenetic inheritance. Indeed, cross-fostering designs show direct effects of maternal care on the behavioral and neuroendocrine responses to stress in their progeny, which supports the existence of an epigenetic mechanism (Liu et al., 1997; Francis et al., 1999a). Weaver et al. (2004) identified modifications in a region that regulates the expression of the coding regions of the glucocorticoid receptor (GR) gene and the differences found in the hippocampus of the offspring from dams exhibiting high and low levels of maternal care could be reversed by cross-fostering (Weaver et al., 2004). An epi-mutation in the GR exon 17 promoter that could explain the long-lasting effect of maternal care is suspected. Such increased hypothalamic methylation decreases GR expression and increases hypothalamic-pituitary-adrenal responsivity in the offspring of dams subjected to gestational stress (Mueller and Bale, 2008). Interestingly, the effects of maternal care on cognitive function in the offspring of dams exhibiting less maternal behavior are reversed by exposure to an enriched environment during the peripubertal period (Bredy et al., 2003; Bredy et al., 2004; Champagne, 2008), implying that the epigenetic regions in the rat brain can be modulated by environmental effects beyond the perinatal period. The concept of ‘transgenerational epigenetic inheritance’ corresponding to the transmission of epigenetic information over several generations is demonstrated when 1) a modification of the environment occurred only for F1 embryos and gametes but a phenotypic effect persists in individuals of the third generation (F3) (Guerrero-Bosagna et al. 2012 in mice and Leroux et al., 2017 in quails), and 2) a male or non-pregnant female adult animal is subjected to specific environmental conditions and changes are seen in the F2 generation or later (Anway et al., 2005 in rats and Braunschweig et al., 2012 in pigs). Although several studies tackle the challenging field of transgenerational epigenetic inheritance, only a few results provide a basis for the inheritance of acquired phenotypes through epigenetic mechanisms. The main focus to date about maternal experience transmission, mainly *via* gestational exposure to various stimuli, involved intergenerational and multigenerational inheritance. Transgenerational epigenetic inheritance studies mediated by female gametes compared to male germ cells appear more challenging since *i)* they imply to wait until the third generation, *ii)* germ cells are more heterogeneous, and *iii)* gametes sampling is much more difficult. In addition, the idea that the paternal experience may have direct implications for the fitness of the offspring is quite recent. Besides the nuclear genome, sperm and semen contain a range of epigenetic elements (small RNAs, chromatin modifications and proteins) that are delivered to the zygote upon fertilization (Rando, 2016). In mammals, the first evidence of transgenerational epigenetic transmission *via* the male germline was observed in rats. Offspring exposed to vinclozolin during the gestational period of gonadal sex determination exhibited reduced fertility and sperm counts, and the effect lasted until the fourth generation (Anway et al., 2005). The DNA methylation of a specific region was shown to be different in the sperm of the third generation of individuals whose ancestors had been exposed (Anway et al., 2005). In livestock, only three studies, performed in pigs and birds, have examined transgenerational epigenetic effects (Braunschweig et al., 2012; Brun et al., 2015; Leroux et al., 2017). In pigs, the offspring of the second generation whose grand-sires were fed with a high diet in methylating micronutrients (methionine and cysteine) showed both lower fat and higher shoulder muscle mass compared with controls (Braunschweig et al., 2012). A difference in DNA methylation in the liver was observed between the control and experimental groups (Braunschweig et al., 2012). In quails, eggs injected with the estrogen genistein resulted in differences in the performances of great-grand offspring for various characters such as behavior and fecundity compared with controls (Leroux et al., 2017). Studies are currently underway to identify the molecular mechanisms that explain the epigenetic modifications involved in the latter study. Thus, all these results report evidences that epigenetic marks are inherited and have an effect on phenotypes.

#### Microbiota Inheritance

Among the other information physically transmitted across generations, the microbiota appears to be one of the easiest to study. The microbiota consists of the symbiotic microbial cells (bacteria, archaea, viruses, and eukaryotic microbes) that reside in and on the bodies of animals.

*In utero*, the mammal digestive tract is practically sterile and the main microbial taxa that reside within the mammalian gut do not develop outside their host (Ley et al., 2006). In livestock, the transmission from one generation to the next of all or part of this microbiota is most likely the result of physical contact between newborns and the dam. Colonization begins at birth, due to contact with the dam’s microbial metacommunity during and after the passage through the birth canal, during suckling and maternal care, and contact with immediate environment (nest material, feed, feces, etc.). Although the optimal time window during which transmission across generations takes place is not known, this is likely to continue from birth to weaning (when suckling ceases) although the composition of the microbiota is stabilized at a later stage when solid feed habits are established [in humans at about 3 years (Yatsunenko et al., 2012); in rabbits at least 15 days after weaning (Combes et al., 2011)]. Indeed, it has been shown that the birth route (vaginal-delivered *vs*. Cesarean section) and suckling type (breast feeding *vs*. artificial formula) impact greatly on the gut microbiota colonization process (Penders et al., 2006). The gut microbial community of fostered rabbits is closer to that of their adoptive mother than that of their biological mother (Abecia et al., 2007; Daft et al., 2015). In pigs, during the suckling period up to day 14, the nursing dam influences the fecal bacterial community which shows progressive changes, with specific bacteria taxa associated with the nursing sow (Bian et al., 2016). Besides parent/nurse-offspring contact, in some species such as pigs (Soave and Brand, 1991), rabbits (Combes et al., 2014), horses (Crowell-Davis and Houpt, 1985), and rats (Galef, 1979), early coprophagia behavior is also likely to play an important role in parent/nurse-to-offspring transmission of the microbiota. It has been demonstrated that, in rabbits, preventing coprophagia delays microbiota maturation (Combes et al., 2014). Microbial metacommunities from the surrounding environment also interfere with the vertical parent-to-offspring transmission of microbiota. For instance, increasing the hygiene of the animals’ environment can alter transmission mechanisms. In pigs, the farming method (outdoor *vs*. building *vs*. in an isolator with antibiotic treatment) results in differences in microbiota composition that persist until after mother-piglet separation (Mulder et al., 2009). Thompson and Holmes (2009) showed that the microbiota composition of piglets separated from their mother at birth and raised on artificial milk was closer for piglets reared in the same pen than with their biological sibs raised in different pens. Finally, the genetics of the host may also have an influence on the microbiota. Several studies have shown that the microbiota of identical (monozygotic) twins are more similar than those of non-identical (dizygotic) twins (Goodrich et al., 2016; Xie et al., 2016). Based on recent microbiome genome wide-association studies, it has been estimated that genetics may explain 5–10% of the variability in bacterial taxa observed between individuals (Hall et al., 2017). These findings suggest that if the microbiota is transferred from one generation to the next by contact between the foster mother and the young she raises, the host's genetics and the rearing environment also play a role in the composition of its microbiota.

The microbiota has tremendous potential to impact the phenotype of its host (Sommer and Bäckhed, 2013; Marchesi et al., 2015). Numerous studies comparing germ-free colonized animals with conventional animals have demonstrated the influence of the microbiota on the phenotype. For example, the transfer of the microbiota of obese mice to germ-free mice induces a weight gain greater than that induced by the transfer of the microbiota from lean mice (Turnbaugh et al., 2006). Similarly, germ-free mice colonized shortly after birth with Rongchang or Yorkshire pig intestinal microbiota develop the same skeletal muscle phenotypes (fiber type and muscle lipid metabolism) as their microbiota donor pigs (Yan et al., 2016). Crosstalk between the microbiota and the innate immune system enables host-mediated tolerance and containment of the microbial community inhabiting the gut. Several defects in germ-free mice, such as GALT (Gut Associated Lymphoid Tissue) development, expression of several antimicrobial peptides and numbers lamina propria T cells, can be adjusted by colonization of the mice with a complex microbiota or specific bacterial species (Muniz et al., 2012). Finally, the microbiota might also influence behavioral characters in line with the brain-gut axis. Fecal microbiota transplantation from depressed patients to microbiota-depleted rats can induce behavioral and physiological features characteristic of depression in the recipient animals, including anhedonia and anxiety-like behaviors, as well as alterations in tryptophan metabolism (Kelly et al., 2016).

### Information Inherited Across Generations Without Physical Transmission

Environmental inheritance refers to the transmission over generations of behavior, culture, and ecological traditions (Anthes et al. 2010). Here the scope will be limited to social learning and conditioning promoted by interactions with conspecifics, and the use of information from the environment, with emphasis on the vertical inheritance of these characters that is more important for the selection of future breeders in comparison with the oblique or horizontal transmissions.

#### Behavioral Inheritance

Parental care leads to parental effects in the offspring and are recognized as a major source of inheritance that significantly contributes to (behavioral) resemblance between parents and offspring (Clutton-Brock, 1991). Inherited parental effects refer to features from the specific environment provided by parents, including (parenting) personality and social skills that are transmitted to the next generation independently of parental genes (Cheverud and Moore, 1994; Kruuk and Hadfield, 2007; Champagne, 2008). For instance, female offspring can learn from, and be influenced during, their youth by the maternal behavior of their dam. They can use such early social learning to raise their own descendants. This non-genetic vertical transmission of behavioral characters from parents to offspring has been demonstrated by cross-fostering designs, notably in rodents (Champagne, 2008), and in sheep (Sanga et al., 2011). A positive association between the preference for polyethylene glycol (PEG) in experienced ewes and their offspring suggests that the dams’ prior experience and preference for PEG influence their offspring’s food choices, leading to transmission of self-medication across generations. When the progeny of mice exhibiting high maternal behavior are nursed by mice exhibiting low maternal behavior, the offspring show low licking patterns and *vice versa*, which demonstrates that an environmental effect mediated by maternal care exists (Francis et al., 1999a; Francis et al., 1999b). Moreover, mutant mice exhibiting disturbed maternal care can transmit this altered behavior to young wild-type females and their descendants over at least two generations (Curley et al., 2008). Thus, the non-genetic intergenerational transmission of maternal care behavior was proven.

#### Cultural Inheritance

A focal individual can benefit from social learning acquired during social interactions and will be influenced by the (individual or group) behavior of the conspecifics it is raised with, i.e. reciprocal influences of individuals sharing a common environment over short or long periods of time. This transmission of social skills and innovations at the group level refers to cultural inheritance [for a review see Danchin and Wagner (2010) and Jablonka and Lamb (2014)]. An innovation is likely to arise when an individual or group is faced with a new challenge for which it currently has no workable solution in its existing behavioral repertoire (Huffman, 1996). This form of social transmission of information enables the dissemination of (new) cultural knowledge, capacities, and traditions of the social group across generations.

In chimpanzees, there are several examples of cultural inheritance. Leaf swallowing to physically expel intestinal parasites seems to have originated from opportunistic feeding by some individuals, and is transmitted as a behavioral tradition (Huffman and Hirata, 2004) and may correspond to an evolutionary adaptation. Stone-play behavior which has no adaptive value is intergenerationally transmitted (Huffman, 1996; Huffman et al., 2010). Social learning is suspected to occur in several livestock species but its demonstration is complicated (for a review see McVey et al., 2018). This is a complex area of research and the question addressed is whether social learning modulates behavioral responses in conspecifics (Boissy and Le Neindre, 1990; Nicol and Pope, 1992). The consequences of this form of social facilitation on performance are seldom investigated. Gibb et al. (2000) observed activity changes but identified no influence of trainer cows (adults which presence aims at facilitating learning and adaptation) on health or performance of newly-weaned calves. Loerch and Fluharty (2000) obtained inconsistent relationships between trials.

#### Ecological Inheritance

Ecological inheritance is the passing on to descendants of inherited resources and conditions through niche construction (Odling-Smee et al., 2003). In livestock species, conversely to wild species, animals do not choose their environment; it depends entirely on the farming system used. Consequently, ecological inheritance could be considered as negligible in livestock species even if this point of view may be questionable for animals raised in outdoor pens and complex environments.

The behavioral/cultural/ecological inherited factors matter due to their favorable impact on other characters expressed in the focal individual, its offspring or, to a lesser extent, in conspecifics. Indeed, a specific behavior expressed by an animal may have positive influence on its health, survival, and/or growth (i.e. preference for PEG). The influence of a specific behavior on characters carried by other individuals occurs when the living environment of the other individuals changes due to the behavior of the focal individual. In that case, the behavior falls into the largely described category of indirect effects [maternal effects if the female behavior modify environment of the offspring (Wolf and Wade, 2009)] in its mode of action but keep its specificity of behavioral/cultural inheritance due to its mode of transmission (*via* learning). In pig, influence of the inherited maternal behavior on the growth performance and survival of the offspring has been evidenced in several studies (Valros et al., 2002; Rydhmer and Canario, 2014; Ocepek and Andersen, 2018). The influence of behavior on other characters can also occur *via* alternative mechanisms (for example epigenetic or microbiota). For instance, maternal behavior plays a predominant role in the establishment of personality since it “programs” hypothalamic-pituitary-adrenal responses to stress in the offspring (Liu et al., 1997). Maternal behavior can favor microbiota transmission that will have an impact on the growth and survival performance of the animal that received this microbiota. These influences make behavior/cultural inheritance an important component for breeding improvement.

To sum up, we described four different sources of inheritance that differ according to their mean of transmission. It is important not to confuse the mean of transmission with the mean of action. Such distinction is particularly relevant in the case of indirect effects (Bijma, 2014; Kong et al. 2018). For instance, let’s consider the case of a maternal behavior “A” that has a positive impact on the performance “B” of the offspring raised by the female under study. In other words, there is a maternal effect affecting the performance of the offspring i.e. a causal influence of the maternal phenotype on the offspring phenotype (Wolf and Wade, 2009). This mean of action (maternal/indirect effects) does not provide any indication on the mean of transmission for maternal effect “A” and phenotype “B” expressed in the offspring. If “A” is influenced by genetic and environmental effects, then “A” is genetically inherited. The dam will partly transmit its “A” performance to its genetic offspring but not to its adoptive offspring. On the other hand, if phenotype “A” is expressed by mimicking what the young female experienced during early life only (behavioral inheritance), then in the case of cross-fostering the dam will partly transmit its “A” performance to the offspring it raises but not to its genetic offspring. The effect of “A” on phenotype “B” is the same in both situations (the mean of transmission changed but not the mean of action). Same type of reasoning can be made for epigenetic and microbiota inheritances as well as for the combination of the different sources of inheritance. A phenotype can be inherited *via* different means of transmission.

## How to Use Extended Heritability for Selection in Livestock Species

In livestock, selection is aimed at improving numerous phenotypes in order to increase animal production, facilitate breeding, reduce animal mortality and morbidity, increase animal welfare, etc. Selecting, as parent of the next generation, animals that will transmit their good skills to their offspring for the characters of interest proved to be an efficient way to enhance the above characters. Only genetic values are accounted for selection in livestock. The inclusion of the other sources of inheritance, by predicting the extended (genetic and non-genetic) transmissible value of an individual in the selection of the future reproducers, would be an added value. In addition, since non-genetic inherited factors are sensitive to the environment, amending the animals’ environment to promote non-genetic inherited factors that are transmitted to the next generations is key for improving performances.

### Predict the Extended Transmissible Value of Individuals

For selection in livestock species, knowing the potential that an animal can transmit to its offspring is crucial to select the best animals (i.e. animals with the highest transmissible potential value) as parents for the next generation. This potential can be predicted by analyzing the phenotypic resemblance between relatives using a linear mixed model. The phenotype *y* *_i_* of individual, is decomposed into its different components by: 

(1) $$y_{i}=x_{i}\beta +a_{i}+\mathrm{ep}i_{i}+\mathrm{cul}t_{i}+\mathrm{mi}c_{i}+e_{i}$$

Where **β** is the vector of fixed effects and ***x*_*i*_** the vector that links the fixed effects to the observation of animal *i*. *a* *_i_*, *epi_i_*, and *mic_i_* are the additive genetic, epigenetic, cultural/behavioral, and microbiotal transmitted values of individual *i*, respectively. Distribution of the different transmitted values, under the assumption that the variance of the transmitted effect is constant over generations, is written $t∼N(0,\Sigma_{t}\sigma_{t}^{2})$ where *t* = *a,epi,cult* or *mic* and $\Sigma_{t}\sigma_{t}^{2}$ is the covariance matrix between the value *t* of different individuals. To predict the transmitted values of an individual, the variance components of the aforementioned model have to be estimated. To do so, phenotypic records recorded in a proper design, genealogic information and the distributions (i.e. $\Sigma_{t}$) of the transmitted values are needed. The matrices $\Sigma_{t}$ can be derived from the laws of transmission of information between individuals or by recording additional information about the inherited factors.

In a recent study, David and Ricard (2019) reported the laws of transmission for the different sources of inheritance. The general form of the law of transmission of a heritable information *t* *(t = a,epi,cult* or *mic* can be decomposed as follows for animal *i* born from sire *s* and dam $d:t_{i}=\lambda_{t,s}t_{s}+\lambda_{t,d}t_{d}+\Sigma_{j\in I_{i}}\lambda_{t,j}t_{j}+\varepsilon_{t,i}$ with *t_i_* the value of animal *i* for inherited factor *t*, $\lambda_{t,s}$ and $\lambda_{t,d}$ the sire and dam path coefficients of transmission for inherited factor *t*, $\lambda_{t,j}$ is the path coefficient of transmission from conspecific *j* and *I_i_* is the group of conspecifics that transmit factor to the target animal $i\;\left(0\;\leq \;\lambda_{t,s}\;\leq \;1,0\;\leq \;\lambda_{t,d}\;\leq \;1,0\;\leq \;\lambda_{t,j}\;\leq \;1\;\mathrm{and}\;\lambda_{t,s}\;+\;\lambda_{t,d}\;+\;\Sigma_{j\in I_{i}}\;\lambda_{t,j}\;\leq \;1\right)$ and, under the assumption that the variance of the transmitted effect is constant over generations, $\varepsilon_{t}∼N[0,I(1-\lambda_{{}^{t,s}}^{2}-\lambda_{t,d}^{2}-\Sigma_{j\in I_{i}}\lambda_{t,j}^{2})\sigma_{t}^{2}]$ where $\sigma_{t}^{2}$ is the variance of the transmitted effect *t*. A specific set of $\lambda_{t,s},\lambda_{t,d,},\lambda_{t,j}$ parameters is related to each inherited factor. The path coefficients of the genetic effects are known ($\lambda_{a,s}=\lambda_{a,d}=0.5$ and $\lambda_{a,j}=0$). The path coefficients of transmission of the other inherited factors are not known and have to be estimated (Rq: different constraints can reduce the number of path coefficients to estimate, see David and Ricard, 2019 for details). Estimating the variance of the different transmitted information (genetic, epigenetic, and other), their path coefficients of transmission and thus predicting the different inherited value is theoretically feasible if the matrices $\Sigma_{t}$ are different. Nonetheless, to ensure practical identifiability, a huge amount of data with a specific population structure and a simplified model would be needed (David and Ricard, 2019). To overcome this difficulty, the different inherited values can be combined into a single value in the transmissibility model. This model does not estimate the value for each inherited factor separately but has proven to estimate appropriately the extended transmissible value of individuals which is of interest for selection (David and Ricard, 2019). If one wanted to estimate the transmitted value for each source of inheritance separately, additional measurements would be required in order to compute the $\Sigma_{t}$ matrices instead of estimating their constituent elements (i.e. $\lambda_{t,.}$). Computing the $\Sigma_{t}$ matrices by incorporating additional information has been proposed for different inherited factors.

Genomic information to compute the genomic relationship matrix.

Even if the path coefficients of transmission for the genetic effects are known, additional information may be incorporated in the model for genetic inheritance. The ***A*** relationship matrix based on pedigree reflects the expected Identical-By-Descent relationships (IBD), i.e. the average relationships assuming infinite loci. Using SNP markers, it is possible to compute the “real” IBD relationships matrix (genomic relationship matrix ***G***) which is slightly different given the finite genome size (Hill and Weir, 2011). Different methods have been proposed to compute the ***G*** matrix (also combined with the ***A*** matrix) in order to increase the reliability of the predictions of the transmittable genetic value of individuals (Hayes et al., 2009). It should be noted that molecular genetic information can also be used to perform direct selection on genes or genomic regions that affect characters of interest (Dekkers, 2004).

*Measuring Epigenetic Marks to Compute the Epigenetic Relationship Matrix*. As commonly performed for building genomic matrices in order to highlight resemblances between individuals based on genomic data, we could compute the epigenomic relationship matrices (Thomson et al., 2018). Indeed, high-throughput technologies that quantify the epigenome, especially DNA methylation, are now well established and routinely used. The most common and cheapest molecular tools to evaluate the genome methylation profile remain CpG beadchips. Although this molecular tool is not available for livestock species except cattle, alternative methodologies based on deep sequencing are still possible despite their cost. Hence, determining the methylation pattern in various individuals could be used to build an epigenomic matrix pinpointing similarities between samples, and might help to predict the epigenetic transmitted value of individuals.

*Measuring the Microbiome to Compute the Microbial Relationship Matrix*. As previously exposed, the microbiota consists of the symbiotic microbial cells (bacteria, archaea, viruses and eukaryotic microbes) that reside in and on the bodies of animals (Sender et al., 2016). Bacteria are predominant, and this kingdom has been extensively studied (review in Costello et al (2009)). At a given time, each individual (i.e. host) can be associated with a microbial taxonomical count vector, the length of the vector depends on the taxonomical rank (Phylum, Order, Class, Family, Genus, Species) or the number of OTUs (Operational Taxonomical Units) chosen to describe the host’s microbiota. In mammals, the length size of this vector could be less than 10 for Phyla, but around 500 to more than 1000 at the OTU level (Stackebrandt and Goebel, 1994, Claesson et al. 2009). To compare microbiota among individuals, alpha and beta diversity calculations are undertaken. Alpha diversity indices are built to summarize in a unique value the microbial richness (how many different entities) and the relative abundance of each entity of an ecosystem. The most commonly used are the Shannon index and the InvSimpson index. However these indices do not reflect many of the variations observed in the ecosystem and the use of beta diversity is preferable because it compares individuals using the whole microbial vector by calculating a distance matrix. Among the distance metrics commonly used, Bray-Curtis dissimilarity is the most popular (Ricotta and Podani, 2017). More recently, phylogenetic distance were proposed, weighted or unweighted with the entity’s relative abundance (UniFrac) which allows to take into account the phylogenetic distance of the microbes present in the ecosystem (Lozupone et al., 2011). The matrix of distances between the microbiota of different individuals can then be used to estimate the proportion of phenotypic variance explained by the microbiota and to predict the microbiotal value of individuals.

*Measuring Social Interactions to Compute the Cultural Relationship Matrix*. Danchin et al. (2013) proposed to perform partial cross-fostering to disentangle genetic inheritance from cultural inheritance in mixed model analysis. Nonetheless, it seems unclear how this process would allow to disentangle microbial and cultural inheritance. Measuring the social relationship between individuals and integrating this source of information in modeling using an adjacency matrix (Scott, 2017), that depicts all dyadic encounters between individuals of a population observed within a given time period, could be a solution for dissociating cultural inheritance from the other sources of similarity between individuals.

To our knowledge, no studies have yet integrated all the heritable factors that explain phenotypic variability as described in Eq1. Several have investigated at the most two inherited factors at a time [genetic and epigenetic (Varona et al., 2015), genetic and culture (Danchin et al., 2013), microbiota and genetic (Difford et al., 2018)]. Since all the different inherited factors induce covariances between relatives, omitting one of them in the model leads to remaining confusion with the others and results in misestimated variances and bad prediction of the different transmitted values. Values of the omitted components are partly picked up by the others components included in the model; the breeding values predicted by the animal model thus include the other non-genetic inherited effects influencing the character (David and Ricard, 2019). Studies aimed at quantifying the non-genetic inheritance of characters selected in livestock are rare. Paiva et al. (2018) reported an epigenetic heritability of 0.10 for body weight in meat quails, Difford et al. (2018) estimated that a significant part of the variance for CH4 emission in cattle is explained by genetics and microbiota (0.21 and 0.13, respectively). To our knowledge, the part of variance explained by cultural/behavioral inheritance has never been reported in the livestock literature. This limited work on the quantification of non-genetic inheritance in livestock is probably related to the novelty of the subject for these species and the lack of convenient estimation tools and appropriate data which may change in the (near) future.

### Promote Improvement of Non-Genetic Inherited Factors

**Figure 2:**
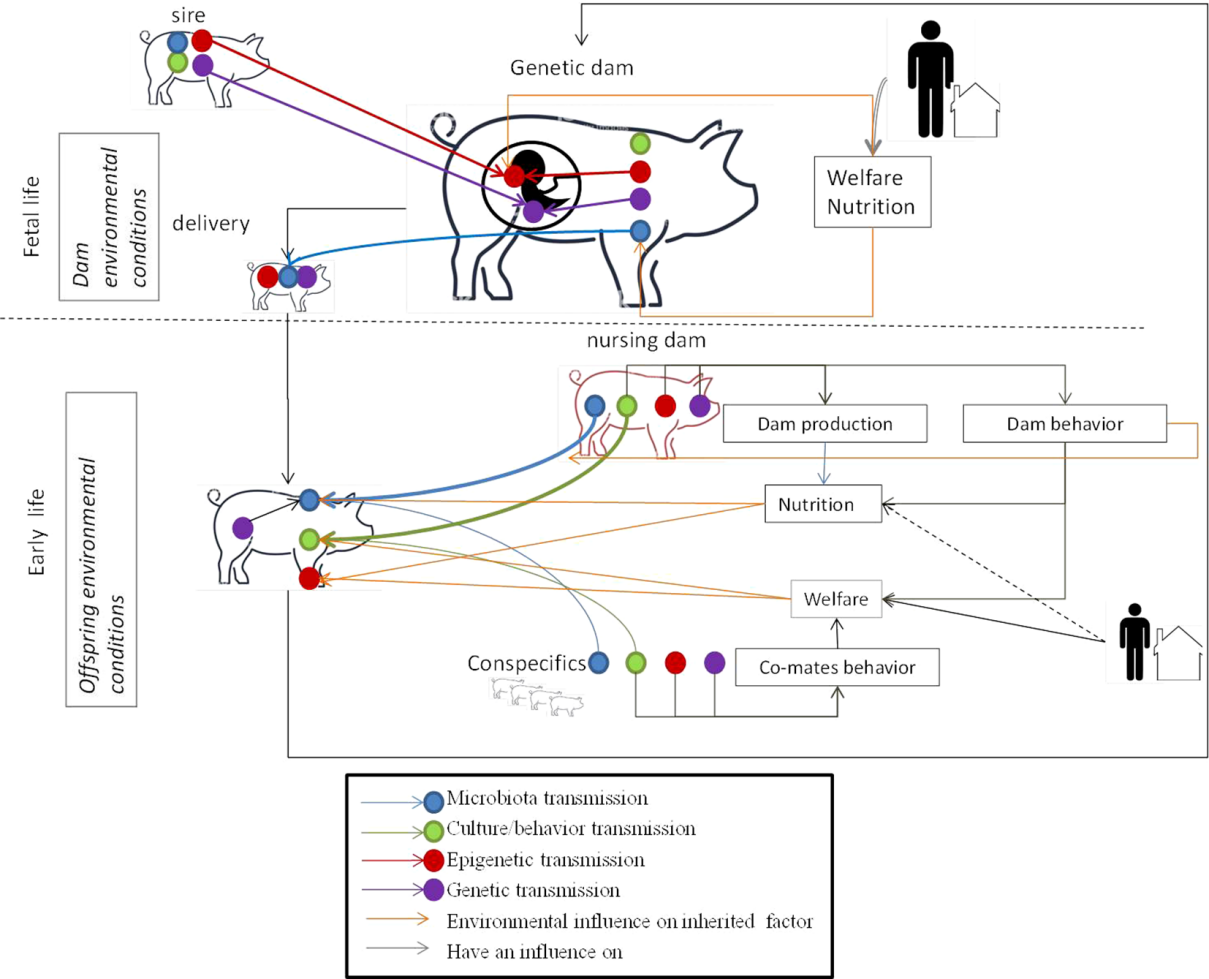
Key moments for the transmission of the different inherited factors and environmental influences during the life of the animal. *Fetal life*: offspring receive DNA and epigenetic marks from their father and mother. The welfare and nutrition of the genetic dam has an influence on the epigenetic marks of the fetus. The housing and nutrition of the dam have an influence on her microbiota. Her microbiota will be transmitted to her offspring during delivery. *Early life*: the young learn culture/behavior from conspecifics (might include the sire in some livestock schemes) and the nursing mother. The welfare of the young influences their behavior. Welfare depends on the breeder by positive contact with the young, housing conditions, and dam behavior which is under genetic, epigenetic, and inherited behavior control. The young share their microbiota with their nursing dam and conspecifics. Microbiota transmission from the nursing dam is facilitated by dam behavior which is under genetic, epigenetic and inherited behavior control. The microbiota is modified by the young animal’s diet, given by the dam for mammalian species, which is under genetic, epigenetic and to a lesser extend behavior control and influenced by the genetics of the young. Epigenetic marks in young animals are modified by welfare and nutrition conditions.

Conversely to the genome, the epigenome, microbiota, and culture are i) influenced by the environment (nutrition, stress, toxicants, maternal care, co-mate interactions, housing conditions, etc.) and ii) dynamic and modulated during the animal’s lifetime. This means that breeders can initiate favorable epigenetic marks, microbiota, or behavior that will be later transmitted across generations by controlling the animal’s environment. It is too early to clearly define the breeding practices to be implemented to influence such factors positively and research is still underway on the subject. However, given current knowledge on non-genetic inherited factors, it is possible to identify different key moments in the lives of the future reproducers when non-genetic inherited factors may be influenced (**Figure 2**).

Environmental factors that have a favorable effect on an individual's phenotype but do not induce changes in the epigenome, microbiota, or behavior that will later be transmitted across generations are not developed in this section because they do not contribute to inclusive heritability.

#### Fetal Life Environment

Fetal life is a key moment for promoting positive epigenetic marks. The epigenetic modifications that occur during fetal development can persist after birth (Vaiserman et al., 2017). Briefly, a wave of genome-wide demethylation followed by a wave of *de novo* methylation successively take place after fertilization except in imprinted regions that resist these changes (Morgan et al., 2005). Particularly, in primordial germ cells (PGCs), the genome undergoes extensive DNA demethylation, including the removal of existing previous parent-specific imprints. New imprints are acquired at later stages of gametogenesis and maintained throughout life, according to the sex of the embryo and with a different timing in the two sexes. In sperm, imprinting starts before birth and is completed during the perinatal period whereas in the female germline, imprints are acquired after birth, during oocyte growth. Thus, given the lability of epigenetic marks, any environmental factors may have a direct or indirect effect on the fetus through its mother (Nicholas and Ozanne, 2019). Recently, Greenberg et al. (2017) provided evidence that transcription during an early embryonic timeframe can program a stable epigenetic state with later physiological consequences on postnatal growth. Consequently, it is likely that influencing the mother’s environment to modify the fetus’s epigenome will be used as a tool for improvement in future breeding schemes. To initiate such positive epigenetic marks, the environment of the pregnant female must be as free of stress as possible in terms of welfare, nutrition, housing, and social interactions. During the fetal period, it is also important not to adversely affect the dam’s microbiota. Use of pre- and probiotics during the last third of gestation may improve the dam’s microbiota that will be transmitted to the offspring at delivery (Le Bourgot et al., 2014; Buddington et al., 2010).

#### Early Life Environment

The early life environment is mainly characterized by mother-offspring interactions and peer interactions. As mentioned above, female offspring can learn from the maternal behavior of their nursing dam and use such early social learning to raise their own descendants. In addition, maternal behavior that modulates the growth environment of the offspring might induce epigenetic marks in the offspring and thus promote favorable epigenetic inheritance (Franklin et al. 2010). Finally, good maternal behavior facilitates microbiota transmission. In practice therefore, given the strong impact of maternal behavior on the different inherited factors, it could be of interest, when possible, to identify dams with good maternal abilities and microbiota, and perform cross-fostering for the potential future reproducers (given their genetic potential) as a tool to promote the transmission of “good” microbiota, epigenome, and behavioral skills to the next generations. In addition, the transmission of a beneficial microbiota could be promoted by limited exposure to stress and adequate nutrition of the nursing dam. Once again, pre- and probiotics can be used. Finally, taking advantage of the coprophagia behavior of the young is also a key point to promote effective microbiota transmission and such behavior should not be hindered by the breeder.

Another solution to promote the transmission of behavioral skills and probably microbiota and initiating positive epigenetic marks is communal nesting (CN) for species in which it is possible (Martinez et al. 2015). Indeed, CN was identified as a favorable configuration that enhanced sociality and brain development in mice (Branchi, 2009). CN combines the two different and independent aspects of early-life social environment: mother-offspring interactions and peer interactions. It is associated with a higher degree of maternal behavior compared with conventional settings (Branchi et al., 2006) and such enhanced maternal care appears to be transmitted over at least three generations (Curley et al., 2008). In livestock, legislation promotes the use of common areas for nursing, notably in pigs. The success of group-housing in sows relies on their maternal behavior. Communal nursing promotes piglet development, increases pre-weaning piglet feed intake, and, by inducing lactational ovulation, gives the piglets a beneficial extended lactation period (Van Nieuwamerongen et al., 2014). Of course, these proposals are made under the assumption of herds free of contagious disease. Otherwise, mixing animals is less recommended.

Withdrawal is a critical period for the young animal, especially in terms of stress. The benefits of early social separation and socialization to the development of social skills have been reported in pigs and related to less damage later in life (D’Eath, 2004; Camerlink et al., 2018). But early maternal separation (early weaning) may have drawbacks on social development and result in abnormal behaviors passed on to conspecifics at a later stage (e.g. belly noising in pigs, chewing in calves). Thus, the benefits of weaning time are variable.

In several livestock species (dairy sheep and cows, and poultry), offspring are separated from their mother. Nonetheless, it has been shown that after birth, the mother's absence disrupts microbiota transmission to the offspring and the intestine is colonized by microorganisms from environmental metacommunities (Thompson and Holmes, 2009). Disruption of the mother-offspring link leads to a high incidence of digestive troubles in the young animals. Grouping of young dairy calves, that are isolated from their dam early in life, limits anxiety and favors their proper behavioral development. In addition, it has been postulated that raising these young animals with a dry female should limit the negative impact of mother-offspring link disruption. Moreover any practice that modifies the immediate environment of the young animal also affects the effectiveness of microbiota transmission. Therefore, the use of anti-bacterial desiccant powder, or housing hygiene of the nest or of the maternal pen must be questioned. In poultry, competitive exclusion cultures derived from the caecal contents of domestic fowl are spread on newly hatched chicks, turkey poults, quails and pheasants to protect the young against Salmonella and other enteropathogens (Schneitz, 2005).

Early life is also the time when interactions with the breeder begin. The effect of early experience on temperament, learning ability, and cognition is a complex phenomenon that requires further investigation, and depends upon the major role of stressors. In our opinion, breeders should ensure that first experiences are positive no matter when the handling of animals begins. At a higher level of complexity, sex-specific differences in the intergenerational transmission of non-genetic effects should be considered. Franklin et al. (2011) observed discrepancies between males and females in the social anxiety transmitted to the F2 generation of the MSUS program. Therefore, appropriate reasonable animal handling should be implemented with taking into account the possible differences of behavior between sexes.

#### Later Stages in the Career of the Breeding Animal

Later in the career of the animal, it has been shown that epigenetic mechanisms are key regulators underlying the neuroendocrine control of puberty (Lomniczi et al., 2013). However, it is not known whether this epigenetic control is heritable, and therefore be part of the epigenetic inheritance. Cattle raised in open areas exhibit certain behaviors that are related to their environment, such as bark stripping. This attitude damages trees and is all the more problematic that it is transmitted rapidly through social learning (Gill, 1992). Social learning is important for feed acceptance and coping with novel environments because livestock can be trained to avoid some feeding habits. Learnt aversion can be maintained by mixing groups of trained and naïve animals, so that the conditioning does not vanish by social learning from naïve animals (Nicol, 1995; Wechsler and Lea, 2007). Good animal husbandry practices should be promoted with regard to welfare, pasture and landscape management.

Thus, different actions to promote “positive” non-genetic inherited effects can be implemented at different times during the life of the animal. They can be classified into two main categories: actions that promote transmission to the target animal of “positive” non-genetic inherited factors *via* other animals, and actions that modulate non-genetic inherited factors *via* favorable environmental conditions (**Table 1**). For the first type of actions, breeders should raise individuals considered as potential future reproducers with animals identified as carriers of a “good” microbiota and/or as having “good” behavioral/cultural transmittable skills (nursing or other conspecifics). For the second type of actions, breeders should favor stress-free environments in regard to nutrition, housing conditions, human handling, and interactions with conspecifics.

**Table 1:** Key environmental influences on non-genetic inherited factors.

		Epigenetic	Microbiota	Culture/behavior
Breeder intervention	Antibiotics^1^		–	
	Stress linked to inappropriate handling^2^	–	–	–
Breeding conditions	Continuing mother-offspring link^3^	+	+	+
	Disruption mother-offspring link^4^	–	–	– or +
	Communal nesting^5^	+	+	+
Housing conditions	Circadian rhythm alteration^6^	–		–
	Animal density^7^		–	–
	Temperature^8^	– or +		–
	Over cleaning^9^		–	– or +
Nutrition	Probiotics–Prebiotics^10^		+	– or +
	Underfeeding^11^	–	–	–
	Overfeeding^12^	–	–	– or +

non-exhaustive list of references

^1^Mulder et al., 2009.

^2^Bredy et al., 2003, Val-Laillet et al., 2019, Hemsworth and Coleman, 2010; Hemsworth and Barnett, 2000.

^3, 4^Branchi et al., 2006; Curley et al., 2008; Franklin et al., 2010; Thompson and Holmes, 2009; Abecia et al., 2007; Daft et al., 2015; Bian et al., 2016.

^5^Branchi, 2009; Van Nieuwamerongen et al., 2014; Martinez et al., 2015.

^6^Oh et al., 2018; Kjaer and Vestergaard, 1999; Moinard et al., 2001; Manser, 1996.

^7^Guardia et al., 2011; Marchewka et al., 2013; Cronin et al., 2014.

^8^Abe et al., 2018; Johnson, 2018; Parois et al., 2018; Schütz et al., 2008.

^9^Schneitz, 2005, Le Floc’h et al., 2014; Combes et al., 2017, Schütz et al., 2019; Renaudeau, 2009.

^10^Le Bourgot et al., 2014; Buddington et al., 2010; Wang et al., 2016.

^11^Murdoch et al., 2016; Le Floc’h et al., 2014; Combes et al., 2017; Lawrence et al., 1993.

^12^Murdoch et al., 2016; Nicholas and Ozanne, 2019; Vasaï; et al., 2014; Van Barneveld, 2013.

## Conclusion

Because of its cumulative and stable effect over time, genetic (genomic) selection will remain the main lever for improving livestock characters. However, recent knowledge about the other sources of inheritance in livestock offer the possibility to favor the transmission of non-genetic inherited factors across generations. Combining genetic and non-genetic inheritance will surely improve the benefit of selection.

## Author Contributions

ID, LC, SC, and JD wrote the manuscript.

## Conflict of Interest

The authors declare that the research was conducted in the absence of any commercial or financial relationships that could be construed as a potential conflict of interest.
